# Supplementary material for: Influenza vaccination uptake and factors influencing vaccination decision among patients with chronic kidney or liver disease
Source: PLoS One. 2021 Apr 13;16(4):e0249785. doi: 10.1371/journal.pone.0249785 (PMC8043408; doi:10.1371/journal.pone.0249785)
Supplement: S1 Table — (DOCX) [file pone.0249785.s001.docx]

**S1 Table. Demographic- and transplant associated parameters and influenza vaccination willingness.**

|  | **Patients willing to receive vaccination in the 2020/2021 season (N=213)** | **Patients refusing influenza vaccination (N=269)** | **p-value** |
| --- | --- | --- | --- |
| Age, Mean±SD | 58.4±13.7 | 54.7±15.4 | 0.007 |
| Female gender, N (%) | 86 (40.4) | 99 (36.9) | 0.442 |
| Marital status, N (%) |  |  |  |
| Single | 52/212 (24.5) | 69/268 (25.7) | 0.76 |
| Married | 122/212 (57.5) | 142/268 (53.0) | 0.32 |
| Divorced/Widowed | 38/212 (17.9) | 57/268 (21.3) | 0.36 |
| Highest school degree, N (%) |  |  |  |
| No school degree | 2/210 (1.0) | 7/267 (2.6) | 0.18 |
| Mandatory school | 31/210 (14.8) | 42/267 (15.7) | 0.77 |
| Vocational training/apprenticeship | 88/210 (41.9) | 123/267 (46.1) | 0.36 |
| High school degree | 43/210 (20.5) | 50/267 (18.7) | 0.63 |
| University or college degree | 38/210 (18.1) | 37/267 (13.9) | 0.21 |
| Transplant-associated parameters |  |  |  |
| Living donor, N (%) | 21/128 (16.4) | 30/140 (21.4) | 0.51 |
| Number of transplantations, median (IQR) | 1 (1-1) | 1 (1-1) | 0.10 |

IQR: interquartile range, N: number
