# Supplementary material for: Influenza vaccination uptake and factors influencing vaccination decision among patients with chronic kidney or liver disease
Source: PLoS One. 2021 Apr 13;16(4):e0249785. doi: 10.1371/journal.pone.0249785 (PMC8043408; doi:10.1371/journal.pone.0249785)
Supplement: Attachment — Submitted filename: EDER_Vaccination_PLOSOne_Response_210227.docx [file pone.0249785.s002.docx]

Vienna, February 27, 2021

**RE: PONE-D-21-00261-R1**

**"Influenza vaccination uptake and factors influencing vaccination decision among patients with chronic kidney or liver disease“.**

Dear Editorial Board of PLOS ONE,

We would like to thank the editors and the reviewer for their prompt and detailed review of our manuscript, and for providing excellent suggestions. We are grateful for the opportunity to submit a revised version of our manuscript and are happy if our manuscript will be included in the influenza collection. We have implemented all suggestions from the editors and the reviewer and performed additional analysis that further strengthened our conclusions on factors influencing influenza vaccination decision in patients with chronic kidney or liver disease.

**The most important changes can be summarized as following:**

- We adapted our statistical methods as according to the reviewers suggestions. Inappropriate correlation analysis were removed and uni- as well as multivariate regression analyses were added.
- The vaccination recommendations issued by the Austrian Health ministry were added.
- We further adapted the methods section by clarifying the process of questionnaire distribution and collection as well as the definition of vaccine proponents.
- We now provide the original questionnaire, a translated version in English and obtained permission to submit the raw data upon request from the data clearing committee of the Medical University of Vienna (the respective certificate was uploaded as Supplementary Material).

We are confident that the scientific merit of our manuscript has greatly benefited from these edits. Below you will find a point-by-point response to the editor’s and reviewers’ comments as well as a revised version of the manuscript (both a clean version and a “marked changes” version).

Please do not hesitate to contact us if any further questions arise. We are looking forward to your evaluation of our revised manuscript.

Kind regards,

Michael Eder, Haris Omic, Heimo Lagler and Bernhard Scheiner

**Editor's comments to Author:**

The manuscript focuses on a potential relevant topic. The study, however, presents several shortcomings that should be addressed before reaching sound conclusions.

**A: We want to thank the Editors for giving us the chance to submit a revised version of our manuscript. Below please find the point-by-point response to the criticisms raised.**

To mention some of them, i) need to elaborate in the Introduction on the recommendations issued by the Austrian Federal Health Ministry;

**A: The recommendations on influenza vaccination issued by the Austrian Federal Health Ministry were included in the Introduction section of the revised manuscript:**

- **Introduction page 5:** …” The majority of infections occurs in children, but severe courses are mainly observed in very young- as well as elderly- or immunocompromised individuals (1). According to the Austrian Federal Health Ministry, influenza vaccination is recommended for all adults and particularly for persons aged >60 years, patients with chronic illnesses or other risk factors as well as health care workers (2). Additionally, influenza vaccination was recently included into the cost-free children vaccination program and is generally recommended in children aged >6 months. Despite these extensive national influenza vaccination recommendations, a comparably low vaccination rate of about 6% (2015/16) in the general Austrian population compared to about 73% in the UK was recently reported” ...

ii) concern about the fact that the study is mentioned as prospective, but it seems a cross-sectional study;

**A: We want to thank the editors for this important comment. Of course, our study design is cross-sectional. Therefore, we changed the respective paragraphs in the revised version of the manuscript (Abstract page 4, Methods section page 6):**

- **Abstract: page 4 “**This cross-sectional, questionnaire-based study recruited CKLD patients managed at three Viennese tertiary care centers between July and October 2020”
- **Methods section: page 6 “**This cross-sectional survey was conducted at three departments at two hospitals in Vienna, Austria. (Department for Nephrology and Department for Gastroenterology and Hepatology, Medical University of Vienna and Department for Nephrology, Clinic Ottakring, Vienna, Austria)

iii) need to clarify how the questionnaires were distributed, anonymized and returned to the researchers;

**A: We extended the information on how the questionnaires were distributed and returned to the researchers. Additionally, we also added a more detailed statement on questionnaire anonymization as shown below:**

- **Methods page 7:** **“**Patients entering the nephrology/hepatology outpatient clinic or dialysis units were screened for study inclusion/exclusion criteria by attending physicians, nurses or non-medical hospital staff. If patients fulfilled inclusion criteria and did not fulfil exclusion criteria, questionnaires not asking for any personal data potentially allowing patient identification (i.e. name or date of birth) were distributed. Patients were asked to answer the written questionnaire alone. Afterwards, patients were instructed to return the anonymously completed questionnaire before leaving the hospital.“

iv) major concern about the applied statistical tests through the manuscript;

**A: We want to thank the editors and the reviewer for their detailed comments on our statistical methods. We absolutely agree and adopted our statistical approach accordingly. Please find the updated sections below:**

- **Methods page 9:** **“**Continuous variables were expressed either as mean ± standard deviation (SD) or median and interquartile range (IQR), as appropriate. Categorical variables are reported as absolute and relative frequencies. For comparison of categorial parameters such as sex, highest school degree or donor type, chi-squared test was used. Normally distributed continuous parameters such as age were compared using unpaired T-test, non-normally distributed parameters were compared with the Mann-Whitney U Test. Distribution of data was evaluated by data visualization using histograms. We further calculated odds ratios via binomial logistic regression to analyze associations between the primary outcome: “willingness to receive influence vaccination in the 2020/2021 season” and reported questionnaire items. Significant associations were then included into a multivariable regression model. A two-sided p-value <0.05 was considered statistically significant. SPSS for Windows, Version 26 (SPSS Inc., Chicago, IL, USA) was used for statistical analyses.”

v) unclear on the basis of which question the authors have considered someone a vaccination proponent;

**A: Patients were considered vaccination proponents if they answered with “yes” to question number 12: “Are you planning to get vaccinated against influenza for the next winter season 2020/2021”. This was clarified in the methods section of the revised manuscript. Additionally, the questionnaire was added as Supplemental Material:**

- **Methods section, page 8: “**The main endpoint was willingness to be vaccinated against influence in the winter season 2020/2021 by asking: “Are you planning to get vaccinated against influenza for the next winter season 2020/2021?”, (questionnaire added as supplemental file).

vi) it would be useful to do a logistic regression for all factors and a multiple logistic regression could as well be considered.

**A: We want to thank the editors and the reviewer for their comments on our statistical methods. We absolutely agree and performed additional regression analyses. Please find the results below.**

**Journal Requirements**

1. Please ensure that your manuscript meets PLOS ONE's style requirements, including those for file naming. The PLOS ONE style templates can be found at

https://journals.plos.org/plosone/s/file?id=wjVg/PLOSOne_formatting_sample_main_body.pdf and

https://journals.plos.org/plosone/s/file?id=ba62/PLOSOne_formatting_sample_title_authors_affiliations.pdf

**A: We want to thank the editors for providing the style requirements, which we followed closely.**

2. Our staff editors have determined that your manuscript is likely within the scope of our Call for Papers on Influenza. This editorial initiative is headed by PLOS ONE Guest Editors Dr. Meagan Deming and Dr. Deshayne Fell. The Collection encompasses research on influenza prevention on every level, including in vitro, translational, behavioral, and clinical studies; disease and immunity modelling; as well as new approaches to influenza prevention.

Additional information can be found on our announcement page: https://collections.plos.org/call-for-papers/influenza/

Currently, your manuscript is included in the group of papers being considered for this call. Please note that being considered for the Collection does not require additional peer review beyond the journal’s standard process and will not delay the publication of your manuscript if it is accepted by PLOS ONE.

We would greatly appreciate your confirmation that you would like your manuscript to be considered for this Collection by indicating this in your next cover letter. If you would prefer to remove your manuscript from collection consideration, please specify this in your cover letter.

**A: We are honoured that our manuscript is considered for the influenza collection and will be happy to have it published within this collection.**

3. Please include additional information regarding the survey or questionnaire used in the study and ensure that you have provided sufficient details that others could replicate the analyses.

For instance, if you developed the survey or questionnaire as part of this study and it is not under a copyright more restrictive than CC-BY, please include a copy, in both the original language and English, as Supporting Information.

If the questionnaire is published, please provide a citation to the (i) questionnaire and/or (ii) original publication associated with the questionnaire.

**A: As outlined in the methods section of the revised manuscript we developed the questionnaire specifically for this study and are happy to include the original version as well as an English version as Supporting Information.**

4. We note that you have indicated that data from this study are available upon request. PLOS only allows data to be available upon request if there are legal or ethical restrictions on sharing data publicly. For more information on unacceptable data access restrictions, please see http://journals.plos.org/plosone/s/data-availability#loc-unacceptable-data-access-restrictions.

In your revised cover letter, please address the following prompts:

a) If there are ethical or legal restrictions on sharing a de-identified data set, please explain them in detail (e.g., data contain potentially sensitive information, data are owned by a third-party organization, etc.) and who has imposed them (e.g., an ethics committee). Please also provide contact information for a data access committee, ethics committee, or other institutional body to which data requests may be sent.

b)	If there are no restrictions, please upload the minimal anonymized data set necessary to replicate your study findings as either Supporting Information files or to a stable, public repository and provide us with the relevant URLs, DOIs, or accession numbers. For a list of acceptable repositories, please see http://journals.plos.org/plosone/s/data-availability#loc-recommended-repositories.

We will update your Data Availability statement on your behalf to reflect the information you provide.

**A: We can understand and appreciate the PLOS ONE data availability strategy. After counselling the responsible data clearing committee (Daten-Clearingstelle der Medizinischen Universität Wien, Spitalgasse 23, 1090 Wien,** [**datenclearing@meduniwien.ac.at**](mailto:datenclearing@meduniwien.ac.at)**, www.meduniwien.ac.at/daten-clearingstelle), we are now able to share the anonymized data set. However, according to the data clearing committee and in line with the information on “Human research participant data and other sensitive data” (obtained from https://journals.plos.org/plosone/s/data-availability), the data may only be shared upon request as this data contains potentially identifying information. We uploaded the respective certificate as Supplementary Material.**

5. Please ensure that you refer to Figures 1, 2 and 3 in your text as, if accepted, production will need this reference to link the reader to each figure.

**A: We now included references to the Figures in the results section of the revised version of the manuscript:**

- **Results, page 11:** Of all 127 patients who received influenza vaccination in the last season, 102 (80.3%) patients underwent influenza vaccination because it was recommended by medical personal (figure 1)”
- **Results, page 12:** Sixty patients (58.8%) followed the recommendations from their general practitioners, 26 patients (25.5%) from their outpatient clinic specialist, 7 patients (6.9%) from external physicians, 19 patients (18.6%) from their nephrologists in the dialysis unit and 5 patients (4.9%) from hospital nurses (figure 2).”
- **Results, page 12**: The most frequent reason was fear of side effects or complications (N=121/32.4%, figure 3).

**Reviewers' Comments:**

**Reviewer #1:**

This is a study on a very relevant topic. Statistical methods should, however, be revised.

**A: We want to thank reviewer #1 for the very detailed evaluation of our manuscript, the helpful comments and suggestions and for acknowledging the importance of this topic. We extensively revised our statistical methods. The revised results are displayed below.**

Minor comments:

Introduction:
1) P5, line 9: is “siloed” the correct word?

**A: No, this word is not commonly used, therefore, we adopted this sentence:**

- **Introduction, page 5:** “… Suggested reasons include the lack of financial reimbursement and social marketing, lack of continuity and separated specialty care within the health care system …”

2) Could you elaborate on the recommendations issued by Austrian Federal Health Ministry? (e.g. which groups, ages)

**A: Below please find the revised section on the recommendations issued by the Austrian Federal Health Ministry, which we also included into the revised version of the manuscript:**

- **Introduction, page 5:** “… The majority of infections occurs in children, but severe courses are mainly observed in very young- as well as elderly- or immunocompromised individuals (1). According to the Austrian Federal Health Ministry, influenza vaccination is recommended for all adults and particularly for persons aged >60 years, patients with chronic illnesses or other risk factors as well as health care workers. Additionally, influenza vaccination was recently included into the cost-free children vaccination program and is generally recommended in children aged >6 months. Despite these extensive national influenza vaccination recommendations, a comparably low vaccination rate of about 6% (2015/16) in the general Austrian population compared to about 73% in the UK was recently reported ...

Materials and methods:
1) It is described that the study is prospective. However, to me it seems more as a cross-sectional study. Please check.

**A: We want to thank the reviewer for this important comment. Of course, our study design is cross-sectional. Therefore, we changed the respective paragraphs in the revised version of the manuscript (Abstract page 4, Methods section page 6):**

- **Abstract: page 4: “**This cross-sectional, questionnaire-based study recruited CKLD patients managed at three Viennese tertiary care centers between July and October 2020”
- **Methods section: page 6: “**This cross-sectional survey was conducted at three departments at two hospitals in Vienna, Austria. (Department for Nephrology and Department for Gastroenterology and Hepatology, Medical University of Vienna and Department for Nephrology, Clinic Ottakring, Vienna, Austria)

2) Could you clarify were the questionnaires distributed, how they were anonymized and how were they returned to the researchers?

**A: We extended the information on how the questionnaires were distributed and returned to the researchers. Additionally, we also added a more detailed statement on questionnaire anonymization as shown below:**

- **Methods, page 7/8: “**Patients entering the nephrology/hepatology outpatient clinic or dialysis units were screened for study inclusion/exclusion criteria by attending physicians, nurses or non-medical hospital staff. If patients fulfilled inclusion criteria and did not fulfil exclusion criteria, questionnaires not asking for any personal data potentially allowing patient identification (i.e. name or date of birth) were distributed. Patients were asked to answer the written questionnaire alone. Afterwards, patients were instructed to return the anonymously completed questionnaire before leaving the hospital.“

3) Peritoneal dialysis patients were not included. Could you add the reason for this?

**A: Peritoneal dialysis patients were not included due to organisatory reasons: contrary to patients at the outpatient clinic or the dialysis unit there was no centrally located reception with the possibility to systematically distribute anonymous questionnaires (and to actively rule out multiple inclusion of the same patients).**

4) If questions were asked about h. influenzae and s. pneumoniae, it would be good to add the Austrian recommendations concerning these pathogens in the introduction.

**A: We agree with reviewer #1 that demonstrating the Austrian recommendations will be helpful for understanding the context of the study. Therefore, we included the following statement to the introduction section of the revised manuscript:**

- **Introduction, page 6:** Similarly, the Austrian Federal Health Ministry recommends, vaccination against s. pneumoniae for all children as well as all adults aged >60 years. Vaccination is further strongly recommended in patients with a high risk for a severe course of disease such as patients with chronic kidney disease, liver cirrhosis as well as patients receiving solid organ transplantation (2). Vaccination against h. influenzae B (HiB) is generally recommended for children as well as for patients with immune deficiencies such as patients with deficiencies in T- and B-cell function (2). Additionally, HiB vaccination is recommended in selected patients receiving solid organ transplantation (3).

Results:
1) ACLD is not described equally in the text (Advanced chronic liver disease) and in footnotes of table 1 (acute on chronic liver disease)

**A: The footnote of table 1 is incorrect and was corrected to “advanced chronic liver disease”.**

2) Could you refer in the text to the figures?

**A: We now included references to the Figures in the results section of the revised version of the manuscript (see comment above).**

3) Could you add a translation of the questionnaire as supplementary material? This will clarify the methodology for the reader.

**A: We want to thank reviewer #1 for this important suggestion and uploaded a translated version of the questionnaire as supplementary material.**

Discussion:
1) It is described that there is an increased willingness to vaccinate. However, willingness was only assessed in the current influenza season. Actual vaccination uptake is not the same as willingness to be vaccinated. It would be good to clarify this.

**A: We absolutely agree with the respected reviewer, checked the whole manuscript for correct wording and adopted the following sections of the manuscript accordingly:**

**Abstract, page 4:**

- **Abstract, page 4:** In total 516 patients (38.1% female, mean age 56.4 years) were included. 43.9% of patients declared their willingness to be vaccinated in the winter season 2020/2021, compared to 25.4% in 2019/2020 and 27.3% in 2016-2018.
- **Abstract, page 4:** Most frequent reasons for not planning vaccination were fear of a) graft injury (33.3%), b) complications after vaccination (32.4%) and c) vaccine inefficiency (15.0%). …”
- **Abstract, page 4:** While influenza vaccination willingness in patients with CKLD is increasing in the 2020/2021 season, vaccination rates may still remain <50%. …”
- **Results, page 10:** 43.9% of patients declared their willingness to be vaccinated in the winter season 2020/2021, compared to an actual vaccination rate of 25.4% in 2019/2020 and 27.3% in 2016-2018.
- **Results, page 10:** However, only eighty-one patients (16.7%) reported vaccination / willingness for vaccination at all three time points. …”
- **Results, page 10:** Willingness for vaccination was highest in kidney transplant- (47.4%) and lowest in liver transplant recipients (20%) but the difference did not reach statistical significance.
- **Results, page 11:** Declared willingness to receive vaccination was associated with the self-perceived risks of infection and associated complications (p<0.001).

2) It would be good to add in the limitation section that vaccination recall-bias might have played a role and that self-assessed vaccination data might not always be the most appropriate vaccination uptake measure.

**A: We included this important limitation to the discussion section of the revised manuscript:**

- **Discussion, page 16:** “… Our study has some potential limitations. … Furthermore, the main parameter of interest was the influenza vaccinate rate in the upcoming winter season and false statements due to socially expected behavior cannot be excluded. Nevertheless, we tried to minimize this potential effect by completely anonymizing the survey. Additionally, self-assessed vaccination data might not always be the most appropriate vaccination uptake measure, however, due to the anonymous study design, we could not link data derived from the questionnaires to electronic patient records. …”

Major Comments:
I have concerns about the applied statistical tests, this should be checked and clarified throughout the manuscript

1) It is stated in method section that for comparison of continuous data unpaired Student´s t- or Mann-Whitney-U-test, and for correlation analysis Spearman’s Correlation Coefficient were used.
- Could it be clarified which test was used for what?

**A: We agree with the reviewer and revised the statistical methods in our manuscript. We further removed correlation coefficient analysis and included univariable and multivariable logistic regression analysis instead:**

- **Methods, pages 9:** Continuous variables were expressed either as mean ± standard deviation (SD) or median and interquartile range (IQR), as appropriate. Categorical variables are reported as absolute and relative frequencies. For comparison of categorial parameters such as sex, highest school degree or donor type, chi-squared test was used. Normally distributed continuous parameters such as age were compared using unpaired T-test, non-normally distributed parameters were compared with the Mann-Whitney U Test. Distribution of data was evaluated by data visualization using histograms. We further calculated odds ratios via binomial logistic regression to analyze associations between the primary outcome: “willingness to receive influence vaccination in the 2020/2021 season” and reported questionnaire items. Significant associations were then included into a multivariable regression model. A two-sided p-value <0.05 was considered statistically significant. SPSS for Windows, Version 26 (SPSS Inc., Chicago, IL, USA) was used for statistical analyses.

2) In results: “Influenza vaccination in the previous season (2018/2019) as well as in the years before (2016-2018) was significantly associated with the intention to receive influenza vaccination this season (R=0.511, p<0.001 and R=0.440, p<0.001).”

Is this a Spearman’s correlation as described in the methods section? Assuming that both the dependent and independent variable are dichotomous, spearman’s correlation might not be an appropriate measure. Spearman’s correlation is a measure for the association between two continuous or ordinal variables and thus not dichotomous variables. Other tests might be more appropriate (e.g. reporting odd ratio via binomial logistic regression). This also counts for assessment of other associations later mentioned in result section. Could this be checked?

**A: We want to thank the reviewer for this important comment. We absolutely agree and adopted our statistical methods accordingly (as outlined above):**

**Abstract**

- **Page 4:** Vaccination uptake was associated with the self-perceived risks of infection (OR: 2.8 (95%CI: 1.8-4.5), p<0.001) and associated complications (OR: 3.8 (95%CI: 2.3-6.3), p<0.001) as well as with previously received influenza vaccination (2019/2020: OR 17.1 (95%CI: 9.5-30.7), p<0.001; season 2016-2018: OR 8.9 (95%CI: 5.5-14.5), p<0.001). Most frequent reasons for not planning vaccination were fear of a) graft injury (33.3%), b) complications after vaccination (32.4%) and c) vaccine inefficiency (15.0%).

**Results**

- **Page 10**: “… Patients who received influenza vaccination in the previous seasons were significantly more likely to declare their willingness to take influenza vaccination in the upcoming winter season 2020/2021 (odds ratio (OR) for patients who received influenza vaccination during the season 2019/2020: 17.1 (95%CI: 9.5-30.7), p<0.001; season 2016-2018: OR 8.9 (95%CI: 5.5-14.5), p<0.001). …”
- **Page 11**: “… Older age (per 10 years: OR: 0.9 (95%CI: 0.8-1.2), p=0.856) was not associated with pneumococcal vaccination status. …”
- **Page 12**: “ … Patients believing to have an increased risk for influenza infection, were almost three times more likely (OR: 2.8 (95%CI: 1.8-4.5), p<0.001) to be willing to receive influenza vaccination. In line, patients stating to have an increased risk for influenza associated complications were four times more likely (OR: 3.8 (95%CI: 2.3-6.3), p<0.001) to accept influenza vaccination. …”

**Discussion**

- **Page 14:** “… We hypothesize that the COVID-19 pandemic might be the primary reason for the increasing willingness to receive influenza vaccination. Almost 30% of patients believed that influenza vaccination was becoming more important since the outbreak of the pandemic and this factor remained a significant predictor for vaccination willingness in multivariable regression analysis. We also found that patient´s individual risk perception and their previous vaccination behavior were strong indicators for the plan to receive vaccination. …”

3) In results: “When comparing between different cohorts, kidney transplant recipients had the highest rate of influenza vaccination proponents (47.4%) compared to patients with CKD not requiring kidney replacement therapy (42.9%), patients receiving HD (43.5%) and patients with ACLD (29.3%).”

- Did you assess the differences between the cohorts statistically?

**A: We compared the cohorts by using chi-squared test, however, the difference did not attain statistical significance. We added this information accordingly:**

- **Results section, page 10:** Willingness for vaccination was highest in kidney transplant- (47.4%) and lowest in liver transplant recipients (20%). The vaccination willingness did not differ significantly between all five groups (p=0.25).

- Could you add in the methods on the basis of which question you consider someone a vaccination proponent?

**A: Patients were considered vaccination proponents if they answered with “yes” to question number 12: “Are you planning to get vaccinated against influenza for the next winter season 2020/2021”. This was clarified in the methods section of the revised manuscript. Additionally, the questionnaire was added as Supplemental Material:**

- **Methods section, page 8:** “… The main endpoint was willingness to be vaccinated against influence in the winter season 2020/2021 by asking: “Are you planning to get vaccinated against influenza for the next winter season 2020/2021? (as demonstrated in the questionnaire added as supplemental file)”

4) In results: “Patients planning influenza vaccination were older [59.5/50.0-68.0 years vs. 56.0/44.0-66.0 years (median/IQR), p=0.024] and more often born in Austria (47.8% vs. 30.6%, p=0.009).”

- Which test did you use for assessment of ‘born in Austria’. This is not a continuous variable and tests for non-continuous variables are not described in method section.

**A: We thank the reviewer for the remark. This finding was analysed by chi-squared test. As outlined above, we adapted the ‘statistics’ part of the Methods section.**

- Maybe it could be checked if it would not be better to do a logistic regression for all factors and a multiple logistic regression could as well be considered.

**A: We agree with reviewer #1 that binary and** **multivariable logistic regression analysis may be better suited to detect parameters associated with the willingness to receive influenza vaccination. Therefore, these analyses were performed, the following sentences were added and the table below was included into the revised version of the manuscript (Table 2).**

**Results**

- **Page 10**: “Patients who received influenza vaccination in the previous seasons were significantly more likely to declare their willingness to take influenza vaccination in the upcoming winter season 2020/2021 (odds ratio (OR) for patients who received influenza vaccination during the season 2019/2020: 17.1 (95%CI: 9.5-30.7), p<0.001; season 2016-2018: OR 8.9 (95%CI: 5.5-14.5), p<0.001).
- **Page 10/11: “**Other sociodemographic- [female gender: OR: 1.2 (95%CI: 0.8-1.7), p=0.442, marital status: married compared to single: OR: 1.1 (95%CI: 0.7-1.8), p=0.554; divorced/widowed compared to single: OR: 0.9 (95%CI: 0.5-1.5), p=0.660), level of education (as displayed in Table 2)] and transplant-associated parameters [deceased donor: OR: 0.8 (95%CI: 0.5-1.5), p=0.482; number of transplantations: OR: 0.8 (95%CI: 0.5-1.4), p=0.413] did not change the likelihood of vaccination willingness.
- **Page 13**: “Next, we performed multivariable analysis by including variables with statistically significant results in univariable regression analyses into a multivariable model (table 2). While age [per 10 years: OR: 0.8 (95%CI: 0.7-1.0), p=0.064] and a higher self-perceived risk for infection [OR: 1.5 (95%CI: 0.6-3.6), p=0.364] did not attain statistical significance, previous vaccinations [2019/2020: OR: 20.3 (95%CI: 7.9-52.0), p<0.001 and 2016-2018: OR: 3.9 (95%CI: 1.7-9.1), p=0.002] as well as a higher risk for a severe course of disease [OR: 2.4 (95%CI: 1.0-5.8), p=0.046] were independently associated with vaccination willingness. Importantly, a change of opinion due to COVID-19 pandemic resulted in the highest odds ratio for vaccination willingness [OR: 23.1 (95%CI: 10.9-49.1), p<0.001].”

**Table 2:**

| Parameters | Univariable | | Multivariable | |
| --- | --- | --- | --- | --- |
|  | HR (95% CI) | P-value | HR (95% CI) | P value |
| Age, per 10 years | 1.2 (1.0-1.3) | 0.008 | 0.8 (0.7-1.0) | 0.064 |
| Female | 1.2 (0.8-1.7) | 0.442 | - |  |
| Marital status |  |  | - |  |
| Single | 1 |  |  |  |
| Married | 1.1 (0.7-1.8) | 0.554 |  |  |
| Divorced/Widowed | 0.9 (0.5-1.5) | 0.660 |  |  |
| Highest school degree |  |  | - |  |
| No school degree | 1 |  |  |  |
| Mandatory school | 2.6 (0.5-13.3) | 0.256 |  |  |
| Vocational training/apprenticeship | 2.5 (0.5-12.3) | 0.259 |  |  |
| High school degree | 3.0 (0.6-15.3) | 0.183 |  |  |
| University or college degree | 3.6 (0.7-18.5) | 0.125 |  |  |
| Other | 3.5 (0.5-22.3) | 0.185 |  |  |
| Previous influenza vaccinations |  |  |  |  |
| Vaccination 2019/2020 | 17.1 (9.5-30.7) | <0.001 | 20.3 (7.9-52.0) | <0.001 |
| Vaccination 2016-2018 | 8.9 (5.5-14.5) | <0.001 | 3.9 (1.7-9.1) | 0.002 |
| Transplantation-associated parameters |  |  | - |  |
| Deceased donor | 0.8 (0.5-1.5) | 0.482 |  |  |
| Number of transplantations | 0.8 (0.5-1.4) | 0.413 |  |  |
| Self-assessed risk |  |  |  |  |
| Higher risk for influenza infection | 2.8 (1.8-4.5) | <0.001 | 1.5 (0.6-3.6) | 0.364 |
| Higher risk for severe influenza course | 3.8 (2.3-6.3) | <0.001 | 2.4 (1.0-5.8) | 0.046 |
| Change of opinion due to COVID-19 pandemic |  |  |  |  |
| Yes | 4.8 (3.0-7.5) | <0.001 | 23.1 (10.9-49.1) | <0.001 |

5) In results “The proportion of patients reporting these concerns was highest in kidney transplant recipients (83.3% and 82.1%) and differed across the groups (patients with CKD not receiving kidney replacement therapy 57.6% and 56.0%, patients receiving hemodialysis 62.5% and 64%, liver transplant recipients 50% and 62.5%, patients with ACLD 55.3% and 50%; p<0.001 for both risks).”

- It is not clear which test was used here and if it is determined where the difference lies.

**A: We thank the reviewer for the remark. This finding was analysed by chi-squared test. As mentioned above we adapted the statistical methods accordingly.**

6) In results: “This specific reason was reported significantly more frequently from patients with high school degree or university degree (15.3% vs. 8.0%, p=0.012).”

- which statistical test is used here?

**A: We thank the reviewer for the remark. This finding was analysed by chi-squared test. As mentioned above we adapted the statistical methods accordingly.**

7) In results: “While patients not born in Austria reported significantly more often about financial obstacles (16.3% vs. 4.8%; p<0.001), profound skepticisms regarding the success of the vaccination was significantly more frequent in patients born in Austria (4.3% vs. 18.8%, p=0.003). Patients who reported financial expenses as reasons against vaccination were significantly younger when compared to those who did not mention the financial burden of vaccination [48/36-60 years vs. 58/48-67 years (median/IQR); p=0.005].”

- which statistical test is used here?

**A: We thank the reviewer for the critical comment. In this analysis we compared the number of patients who indicated a financial obstacle between patients born in Austria and patients born abroad. We therefore used chi-squared test.**

**References**

1.	Kumar D, Michaels MG, Morris MI, Green M, Avery RK, Liu C, et al. Outcomes from pandemic influenza A H1N1 infection in recipients of solid-organ transplants: a multicentre cohort study. Lancet Infect Dis. 2010;10(8):521-6.

2.	Bundesministerium für Soziales G, Pflege und Konsumentenschutz. Impfplan Österreich 2021. 2021.

3.	Duchini A, Goss JA, Karpen S, Pockros PJ. Vaccinations for adult solid-organ transplant recipients: current recommendations and protocols. Clinical microbiology reviews. 2003;16(3):357-64.
